# Groundwater inputs could be a significant but often overlooked source of phosphorus in lake ecosystems

**DOI:** 10.1038/s41598-024-66985-z

**Published:** 2024-07-15

**Authors:** M. Sol Lisboa, Rebecca L. Schneider, Lars G. Rudstam, M. Todd Walter

**Affiliations:** 1 Department of Biological and Environmental Engineering, Cornell University, Ithaca, NY 14853 USA; 2 Department of Natural Resources and the Environment, Cornell University, Ithaca, NY 14853 USA

**Keywords:** Element cycles, Hydrology, Environmental impact, Limnology

## Abstract

Freshwater lakes are severely threatened, due largely to excess inputs of nutrients and other contaminants. Phosphorus (P) is receiving renewed attention due to recent increases in toxic cyanobacteria blooms in lakes worldwide. We investigated groundwater seepage for its role in P loading dynamics at Oneida Lake, New York, USA—one of the most well-studied lakes globally. P loading was measured at representative sites along the 88 km shoreline over three summers by directly measuring groundwater flow using seepage meters and porewater samplers. Groundwater seepage was a continuous and significant source of dissolved P over the summer months, comparable to tributary sources to the lake during that time. This constant input has enriched the concentrations of P in the nearshore surface waters, significantly above levels in the pelagic zone. Pore Total Phosphorus (TP) concentrations and loads reached extremely high values (up to 100 mg/L), with inorganic P representing only ~ 10% of TP per site. Groundwater seepage flows and P loadings were highly variable across space and time, partially explained by adjacent land uses and precipitation. Our research concludes that groundwater seepage is a significant, but overlooked, source of dissolved P and a crucial factor driving summer primary production at Oneida Lake, and likely other temperate lakes.

## Introduction

Excess loading of nutrients is one of the biggest threats to lake water quality and ecosystems, despite ubiquitous, long-term efforts to reduce nutrient inputs from the surrounding landscapes^1^. In general, phosphorus (P) is considered the nutrient limiting primary production in temperate lakes and drives nuisance algal growth^2,3^. Since the 1970’s, P loading into several lakes has been significantly reduced with the implementation of water quality agreements and the construction of sewage treatment plants, although agricultural P pollution is still significant in some locations. Renewed attention to P loading has risen globally due to the increase in often toxic, blue-green algal blooms (Hazardous Algal Blooms or HABs). The synergistic, detrimental impacts of HABs on lake ecosystems, human health, and aesthetics result in significant financial costs to local and regional economies by impairing water quality for drinking water, recreation, fishing and other uses^4,5^.

In order to conduct effective nutrient management, it is important to quantify and evaluate the contribution of the various P sources to lakes. The main pathways include precipitation, surface water discharge, overland stormwater runoff, and groundwater seepage. However, groundwater loading into lakes has been seriously overlooked^6,7^. Indeed, the linkage between a lake and its adjacent groundwater is still one of the least studied factors in lake hydrology and ecology^8,9^ with hydrologic studies limited to ~ 100 different lakes globally as of 2015^10^. River-groundwater interfaces, (i.e. hyporheic zones)^11,12^ and the marine-groundwater interface have been much better studied^13–15^.

Groundwater contributions to a lake’s nutrient budget are difficult to quantify and characterize, due in part to the high temporal and spatial heterogeneity of its fluxes. In addition, as water flows across the sediment–water interface, its chemistry undergoes rapid shifts in biogeochemical conditions including temperature, pressure, organic matter content, biological activity, and oxygen concentration^16,17^. As a result, direct groundwater nutrient loading estimates are scarce, and estimations based on groundwater composition in nearby wells can be misleading^18,19^. In addition, P has not been traditionally considered a major component of groundwater due to its tendency to bind to sediments and soil particles. As a result, P contributions to lake systems by groundwater seepage have been rarely studied, except in a few small lakes^20,21^.

However, there are powerful arguments supporting a larger role for groundwater in the P dynamics of diverse types of lake systems. Although considerable research on P has focused on storm runoff and snowmelt events^22^ these high flow events dominate in winter and early spring in temperate regions, whereas primary productivity, with its associated biological demand for nutrients, peaks in late spring and summer when surface discharge is low and groundwater input may dominate. There is also growing evidence that P can be transported in aquifers by groundwater flow^23,24^. For example, Holman, et al.^25^ study in England found concentrations of soluble reactive phosphate above the regulatory threshold in many areas, with land cover as a significant predictor, and concluded that groundwater contributions to surface water may have the potential to trigger or sustain eutrophication. Groundwater has also been shown to be a significant source of nutrients in naturally oligotrophic pine barren ponds^26^ and in lakes not dominated by groundwater flow inputs^20,27,28^. Along with geochemical conditions, such as redox potential and iron concentrations^29,30^, other factors, such as the sorption potential of the aquifer matrix, the degree of P saturation, and the time of contact between groundwater and the aquifer matrix^21,31,32^, could also greatly influence P concentrations and transport via groundwater.

A consistent finding from the hydrologic studies is that direct discharge of groundwater is concentrated within 50 m from the lake edge, where shallow, local flow paths intersect with lake bottoms. However, most nutrient studies have focused on the pelagic, open waters and these littoral zones have been generally overlooked. Only recently has research centered on understanding shore conditions and ecology more systematically, including the impacts of intensified land use^33–39^. Cumulatively, these studies suggest that offshore areas of many lakes are appearing oligo to mesotrophic, whereas nearshore areas exhibit eutrophic conditions, including elevated nutrient concentrations, nuisance growth of filamentous algae and cyanobacteria blooms. But groundwater seepage continues to be poorly studied for its potential as a source of nutrients and contaminants in the nearshore areas, and its role connecting terrestrial and lake processes. Understanding of the interplay of hydrological, biogeochemical, and socioeconomical factors in these areas is a key component to improve our understanding of nutrient pollution, and to develop effective watershed-based approaches to lake management.

The overall goal of this study was to evaluate groundwater seepage for its role in the P loading dynamics along the entire shoreline of Oneida Lake, located in central NY, USA. The lake is shallow, well-mixed (i.e. polymictic), one of the most well-studied mesotrophic lake ecosystems globally and used as model for management of the Great Lakes^40^. At 207 km^2^, it is also one of only a handful of lakes larger than 10 km^2^ where groundwater hydrology has been investigated^41^, and for which tributaries are the primary water source.

Specifically, we aimed to:

1. Quantify and compare groundwater seepage flow rate, P concentrations, and the resulting P loads in Oneida Lake during the summer, both intensively among 13 representative sites located along 800 m of the southern shoreline (2017, 2018) and extensively among 10 sites distributed around the entire 88 km shoreline (2020).
2. Evaluate potential drivers of groundwater patterns by using statistical models to (a) assess the influence of precipitation prior to sampling events, (b) compare groundwater dynamics among the northern and southern shorelines associated with their large (~ 1700 km^2^) drainage basins, and (c) compare P loading among shorelines differing in proximate adjacent land use (intensive study: Residential, Field, Wetland; extensive study: Forest, Residential, Mixed).

## Results

### Intensive, southern shoreline study

A total of 384 flow measurements were made at the 13 sites during the intensive study. Overall mean groundwater flow rate during the 2017 and 2018 summers along Oneida Lake’s southern shore was 1.35 L/m^2^-day (SD = 3.10), with individual values ranging from − 1.6 to 26.9 L/m^2^-day (Table 1). Negative seepage values were infrequent but indicated recharging groundwater. Mean porewater concentrations of TP averaged 2.11 mg/L and ranged between 0.08 and 25.93 mg/L. These were significantly higher than average SRP concentration which was 0.17 mg/L (SD = 0.34), with the highest value reaching up to 2.22 mg/L. Combining seepage flows and P concentrations, TP loads across all sites was 1.31 mg/m^2^-day (SD = 3.15), with individual values ranging from − 2.91 to 20.67 mg/m^2^-day. SRP loads also exhibited broad variability ranging from − 0.08 to 2.41 mg/m^2^-day at individual sites and averaged at 0.12 mg/m^2^ day (SD = 0.36) (Table 1).

**Table 1:** Groundwater flow, Porewater Total and Soluble Reactive Phosphorus concentrations (mg/L), and Loads (mg/m^2^ day) (mean, standard deviation, minimum and maximum) at Oneida Lake during summer 2017 & 2018 for each land use.

Parameter	Year	2018			
	Category	Field	Residential	Wetland	Total
TP mg/L	n	55	58	47	160
	Mean	2.26	2.63	1.31	2.11
	SD	3.57	3.03	1.38	2.91
	Min	0.11	0.08	0.10	0.08
	Max	25.93	18.75	6.00	25.93
SRP mg/L	n	59	61	58	178
	Mean	0.12	0.31	0.09	0.17
	SD	0.13	0.53	0.12	0.34
	Min	0.004	0.010	0.005	0.004
	Max	0.83	2.22	0.93	2.22
TP Load mg/m^2^ Day	n	55	58	47	160
	Mean	1.80	1.92	0.31	1.31
	SD	3.57	4.01	0.66	3.15
	Min	− 0.12	− 2.91	− 0.23	− 2.91
	Max	16.41	20.67	3.14	20.67
SRP Load mg/m^2^ Day	n	59	61	58	178
	Mean	0.19	0.17	0.03	0.12
	SD	0.47	0.40	0.09	0.36
	Min	− 0.08	− 0.04	− 0.03	− 0.08
	Max	2.40	2.41	0.64	2.41
Flow L/m^2^ Day	n	72	67	75	214
	Mean	1.50	1.19	0.49	1.05
	SD	2.35	3.04	2.03	2.52
	Min	− 1.20	− 0.55	− 1.64	− 1.64
	Max	10.40	23.07	16.12	23.07

	Year	2017			
	Category	Field	Residential	Wetland	Total
Flow L/m^2^ Day	n	109	37	24	170
	Mean	1.95	1.39	1.21	1.73
	SD	3.40	4.92	2.55	3.68
	Min	− 1.48	− 1.05	− 0.89	− 1.48
	Max	22.62	26.90	11.45	26.90

	Year	2017 & 2018 combined			
	Category	Field	Residential	Wetland	Total
Flow L/m^2^ Day	n	181	104	99	384
	Mean	1.77	1.26	0.67	1.35
	SD	3.03	3.80	2.18	3.10
	Min	− 1.48	− 1.05	− 1.64	− 1.64
	Max	22.62	26.90	16.12	26.90

During summer 2018, the average TP and SRP concentrations in the lake’s surface water were significantly lower than pore water concentrations (p-value < 0.001). However, surface water concentrations at near shore areas were one to two orders of magnitude higher than lake water concentrations in the deep offshore areas ~ 2 km away from shore^42^ (Table 2). Dissolved Oxygen in pore water samples ranged from 0 to 6.8 mg/L, with more than 90% of the samples exhibiting DO values below the biological limit of 4 mg/L^43^. Dissolved oxygen concentrations were significantly higher in lake surface samples (mean 9.73 mg/L) than in pore samples at all sites (p < 0.001). Pore DO concentrations were not significantly correlated with pore TP or SRP concentrations^44^.

**Table 2:** Descriptive summary of Total Phosphorus (TP) and Soluble Reactive Phosphorus (SRP) concentrations for lake and porewater samples for the intensive (2018) and extensive (2020) study.

Parameter	Year	Intensive (2018)			Extensive (2020)		
	Summary						
	Category	*Lake (Off Shore)	Lake (Near Shore)	Pore	**Lake (Off Shore)	Lake (Near Shore)	Pore
TP mg/L	n	8	173	160	13	79	87
	Mean	0.009	0.31	2.11	0.015	0.114	25.2
	SD	0.003	1.17	2.91	0.004	0.160	29.02
	Min	0.005	0.04	0.08	0.009	0.009	0.17
	Max	0.015	10.20	25.9	0.022	1.138	102
SRP mg/L	n	8	180	178	13	79	87
	Mean	0.001	0.05	0.17	0.001	0.026	0.092
	SD	0.0003	0.03	0.34	0.002	0.016	0.101
	Min	0.001	0.0003	0.0044	0.001	0.004	0.013
	Max	0.002	0.13	2.22	0.006	0.097	0.846

*Average over summer months during 2018 of the composite samples across 4 open water stations at Oneida Lake (Rudstam, 2021).

**Average over summer months during 2020 of the composite samples across 4 open water stations at Oneida Lake (Rudstam, 2021).

Linear Mixed Models (LMM) indicated that groundwater seepage flow rates differed significantly among the three land uses (p < 0.001). Field sites exhibited significantly higher flows compared to Residential and Wetland areas. Precipitation volume in the previous 24 h. was marginally correlated with groundwater flow rate (p = 0.06) and was independent of land use type. For p concentrations, there was a significant seasonal effect, such that sampling week was positively correlated with both TP concentrations (p < 0.001) and loads (p < 0.05), however none of the explanatory variables were correlated with SRP concentrations. Land uses exhibited significant influences on TP loads (p = 0.008), with Wetlands contributing significantly lower loads than Field and Residential areas. SRP loads were highly correlated with precipitation in the previous 24 h., with loads predicted to increase as precipitation increased. Overall, land uses had a marginal effect on SRP loads, with Wetland sites exhibiting marginally smaller loads than the Field sites (Supplementary Table S1).

### Extensive, lake-wide study

**Figure 1:**
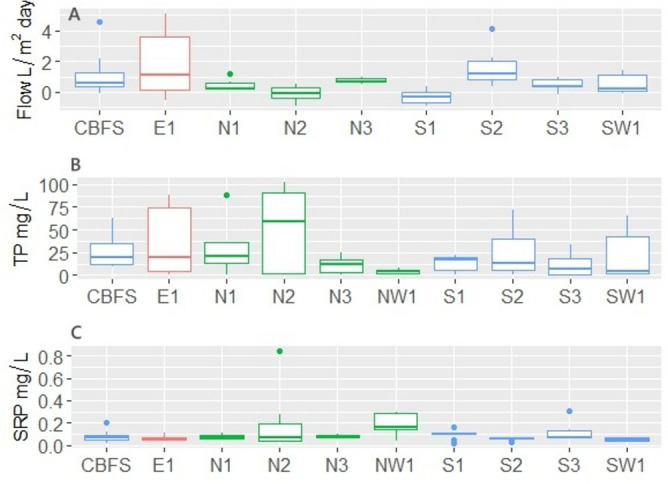
Boxplots showing groundwater flow rates (**A**), Pore TP concentration (**B**), and Pore SRP concentration (**C**) by sampling site during summer 2020.

**Figure 2:**
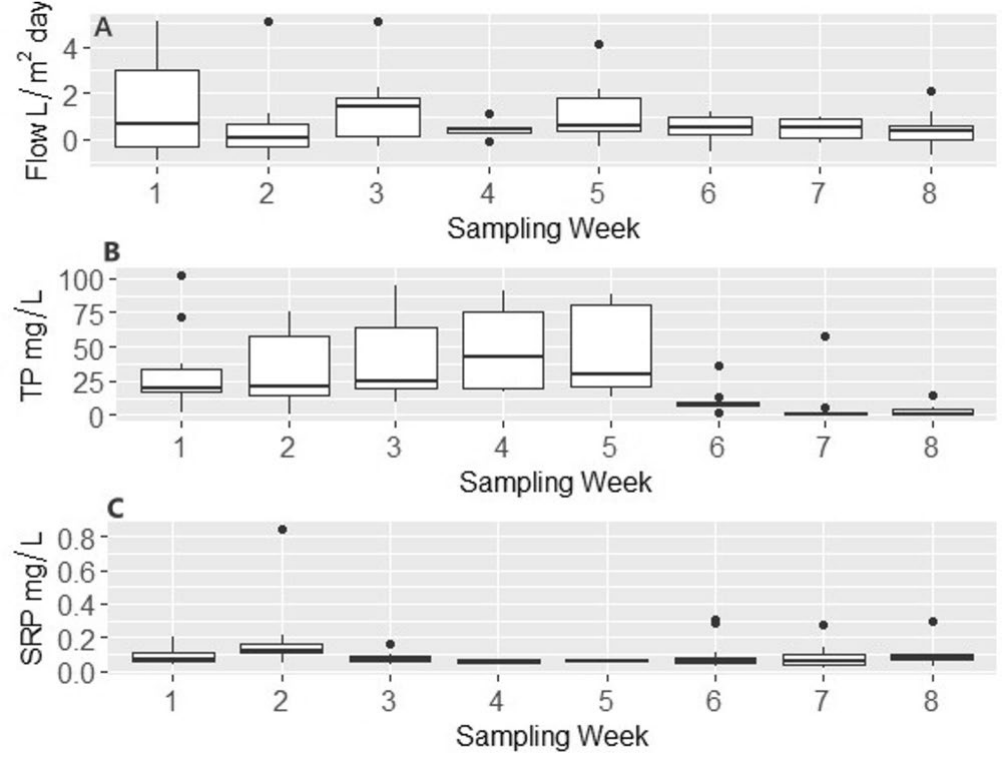
Boxplots showing groundwater flow rates (**A**), Pore TP concentration (**B**), and Pore SRP concentration (**C**) by sampling week during summer 2020.

A total of 87 groundwater seepage flow rates were measured throughout summer 2020 with a mean groundwater flow of 0.81 L/m^2^-day (SD = 1.34) across all sites (Table 3). Recharging groundwater was observed infrequently. Seepage flow rates were highly variable both spatially and temporally (Figs. 1A, 2A). Comparisons between the two shores indicated that average flow rate along the South shore was higher and more variable (mean ± SD = 0.80 ± 1.20 L/m^2^-day) than along the North shore (0.28 ± 0.56 L/m^2^-day) (Table 3, Fig. 1A). Groundwater seepage flow rates were also highly variable throughout the duration of the study period. Some weeks, flow rates were highly different, while on other weeks rates were fairly homogeneous among sites (Fig. 2A).

**Table 3:** Groundwater flow, Pore water Total and Soluble Reactive Phosphorus concentrations (mg/L), and Loads (mg/m^2^ day) (mean, standard deviation, minimum and maximum) by sampling site at Oneida Lake during summer 2020.

Parameter	Summary/site	E1	N1	N2	N3	NW1	S1	S2	S3	S4	SW1	North Shore	South Shore	All
Flow L/m2 Day	n	11	6	10	6	ND	9	11	6	14	9	27	49	87
	Mean	1.92	0.47	− 0.12	0.76		− 0.31	1.54	0.48	1.22	0.57	0.26	0.80	0.77
	SD	2.22	0.40	0.52	0.17		0.44	1.09	0.43	1.55	0.63	0.56	1.20	1.31
	Min	− 0.52	0.16	− 0.93	0.56		− 0.88	0.37	− 0.16	− 0.09	− 0.09	− 0.93	− 0.88	− 0.93
	Max	5.12	1.20	0.51	0.97		0.40	4.16	0.97	4.61	1.41	1.20	4.61	5.12
TP mg/L	n	11	6	10	6	5	9	11	6	14	9	27	49	87
	Mean	34.96	31.67	49.87	11.23	4.47	13.64	23.78	11.53	26.92	21.76	28.73	21.09	25.22
	SD	37.59	34.23	44.96	9.87	3.22	8.62	28.08	13.79	19.88	26.65	36.17	21.50	29.02
	Min	0.88	0.83	0.56	0.24	0.68	0.65	0.17	0.36	9.93	0.69	0.24	0.17	0.17
	Max	88.00	88.43	102.35	24.80	8.67	22.21	72.09	33.78	62.39	64.82	102.35	72.09	102.35
SRP mg/L	n	11	6	10	6	5	9	11	6	14	9	27	49	87
	Mean	0.06	0.07	0.17	0.08	0.18	0.10	0.06	0.12	0.08	0.05	0.13	0.08	0.09
	SD	0.02	0.02	0.25	0.02	0.11	0.05	0.01	0.10	0.05	0.01	0.16	0.05	0.10
	Min	0.04	0.04	0.03	0.06	0.04	0.02	0.02	0.06	0.01	0.04	0.03	0.01	0.01
	Max	0.11	0.11	0.85	0.10	0.29	0.16	0.07	0.30	0.20	0.07	0.85	0.30	0.85
SRP:TP Ratio	n	11	6	10	6	5	9	11	6	14	9	27	49	87
	Mean	0.024	0.025	0.039	0.101	0.108	0.012	0.035	0.087	0.005	0.020	0.064	0.026	0.037
	SD	0.035	0.050	0.070	0.164	0.180	0.008	0.078	0.087	0.005	0.023	0.118	0.054	0.079
	Min	0.0005	0.0007	0.0004	0.0039	0.0201	0.0024	0.0007	0.0021	0.0002	0.0006	0.0004	0.0002	0.0002
	Max	0.083	0.115	0.227	0.401	0.430	0.025	0.259	0.200	0.019	0.057	0.430	0.259	0.430
TP mg/m2 Day	n	11	6	10	6	5	9	11	6	14	9	27	49	87
	Mean	74.84	6.28	4.88	7.57	1.98	0.84	40.73	5.78	31.11	20.40	5.25	22.64	23.85
	SD	133.25	8.23	11.65	6.27	1.93	2.52	51.59	6.43	46.47	33.90	8.47	39.47	58.29
	Min	− 0.06	0.00	− 0.05	0.17	− 0.01	− 0.04	0.19	0.00	− 0.01	0.00	− 0.05	− 0.04	− 0.06
	Max	450.91	22.41	36.34	16.29	4.58	7.56	143.62	13.19	175.12	85.25	36.34	175.12	450.91
SRP mg/m2 Day	n	11	6	10	6	5	9	11	6	14	9	27	49	87
	Mean	0.086	0.035	− 0.002	0.059	0.075	− 0.003	0.092	0.078	0.117	0.029	0.03	0.07	0.06
	SD	0.12	0.04	0.02	0.02	0.07	0.01	0.07	0.11	0.24	0.03	0.05	0.14	0.12
	Min	− 0.016	0.013	− 0.035	0.037	− 0.029	− 0.016	0.008	− 0.003	− 0.002	− 0.003	− 0.03	− 0.002	− 0.002
	Max	0.35	0.11	0.03	0.08	0.15	0.018	0.257	0.294	0.915	0.078	0.15	0.91	0.91

Mean pore TP concentration among all sites around the lake was 25.2 mg/L (SD = 29.02), with individual values ranging from 0.17 to 102 mg/L. Mean pore SRP concentrations were several orders of magnitude lower than pore TP concentrations with an overall mean of 0.09 mg/L (SD = 0.1). As a result, SRP:TP ratio was low with the average ratio per site ranging between 0.005 and 0.11 (Table 3). As was observed in the intensive study, pore water P concentrations were significantly higher than the lake surface water concentrations collected adjacent to the stations along both the North and South shores. Also, surface TP concentrations nearshore were up to two orders of magnitude higher than waters in the offshore area of the lake, while SRP concentrations were an order of magnitude higher in the littoral water compared to open waters^42^ (Table 2). Combining flux rates and concentrations, we documented that the mean TP load across the 10 sites distributed along the entire lake shoreline was 23.9 mg/m^2^-day (SD = 58.3), with individual values ranging from − 0.06 to 450 mg/m^2^-day. SRP loads at individual sites ranged from − 0.002 to 0.91 mg/m^2^-day and averaged at 0.06 mg/m^2^-day (SD = 0.12) (Table 3).

LMM analysis indicated that groundwater seepage flow rates decreased significantly with the numbers of days since the last rain event (p < 0.05). Precipitation amounts in the previous 72 h. was strongly correlated with TP concentration (p < 0.05) and weakly correlated with TP loads (p = 0.09). In contrast, although SRP concentrations were not influenced by precipitation or the number of days since the last rain event, SRP loads were significantly correlated with numbers of days since last rain event (p < 0.05) (Supplementary Table S2, Fig. S1). Unlike the intensive study, the average weekly TP concentrations showed a different seasonal pattern increasing at the beginning of the season (weeks 1–5) and decreasing dramatically later in the summer (week 6–8) (Fig. 2B). SRP concentrations were more homogenous both spatially and temporally (Figs. 1C, 2C).

**Figure 3:**
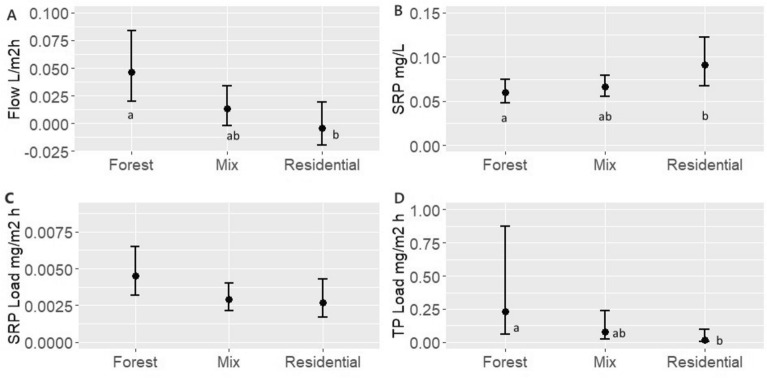
Flow, concentrations, and loads estimates and 95% CI for land use effect. Letters show significant differences between land uses categories (p < 0.05). (**A**) Land use effect on groundwater flow rates. (**B**) Land use effect on SRP concentrations. (**C**) Land use effect on TP loads. (**D**) Land use effect on SRP loads.

Spatially, land use was shown to have a significant effect on all the assessed parameters, except for TP concentrations (Supplementary Table S2). Groundwater seepage flow rates were significantly higher in sites located adjacent to Forested areas compared to Residential sites (p < 0.05). Conversely, SRP concentrations were significantly higher in Residential sites as compared to Forested areas (p < 0.05). Interestingly, Forested sites were associated with higher TP loads than Residential sites (p < 0.05), while SRP Loads exhibited no differences between Forested and Residential sites. Sites with land use classified as Mix (which have components of both Forest and Residential areas) were intermediate between Residential and Forest sites and not statistically different from either of those site classes (Fig. 3).

## Discussion

Three years of studying groundwater processes around the Oneida Lake shoreline confirmed that groundwater is a major, on-going source of dissolved phosphorus to this ecosystem. It influences the entire lakeshore littoral zone, elevating the P concentrations at both the sediment–water interface and in the entire overlying water column, and magnifying the dichotomy between the littoral and pelagic environments. This P loading represents new P originating from an external source in the watershed and is comparable in magnitude to loads from Oneida Lake tributaries^44^ and to internal loading estimates from regional lakes^45,46^. SRP concentrations and loads, although low, remained relatively constant throughout each summer season, indicating that groundwater serves as a consistent source of the readily available, mineral-based P to the littoral environment. However, TP concentrations averaged tenfold higher than that of SRP, with concentrations and loads that were more variable temporally and spatially. Few HAB studies have considered TP partly because P within the algae can be a significant proportion of TP in the water column, and thus not an independent measure for nutrient availability for algal growth. But because cyanobacteria can utilize organic P when inorganic P is scarce^47^, considering dissolved organic P loading is important. This introduces an exciting new hypothesis about groundwater as a previously unrecognized HAB driver. Local and regional precipitation were positively correlated with flow rates and P fluxes. Shore adjacent land use was also a significant driver of water fluxes and P concentrations. Sites adjacent to residential areas exhibited higher P concentrations, possibly due to septic systems or fertilizers. However, the higher TP loads occurred adjacent to forested landscapes as well, and with increasing rainfall, which was unexpected. One possible explanation is that dissolved organic compounds are leaching from the forest soils, such as been proposed for the global phenomenon of lake “browning”^48^. The results of this study suggest that groundwater seepage P loadings may be more significant for lake ecology than traditionally thought, especially in the context of harmful algal blooms and nearshore benthification processes.

Rates of groundwater flow were comparable to those documented previously along Oneida Lake’s shorelines, and to values reported for other lakes. Subsurface flow averaged 1.35 L/m^2^-day of into Oneida Lake South Shore during summer 2018, and 0.81 L/m^2^-day across ten sites located along the entire shoreline during summer 2020. These rates are well within the range that was reported by Schneider, et al.^41^ for 29 sites located around the entire shoreline during summer 1999. These rates are also highly comparable to other studies^20,49^, although both much higher^50,51^, and much lower rates have been reported^52^. Rosenberry, et al.^10^ compiled data for 108 lakes around the world and reported seepage rates ranging from 0.05 to 1140 L/m^2^ day, with a mean of 7.4 L/m^2^ day (Table 4).

**Table 4:** Summary of groundwater flow, pore P concentrations and P Loads into temperate lakes reported in the literature using similar estimation and sampling methods as in this study. SRP refers to Soluble Reactive Phosphorus, TDP refers to Total Dissolved Phosphorus.

Location	References	Average flow (L/m2 day)	Trophic state	Pore P concentration mg/L*	P Load mg m2 day*	Sampling details**
Sparkling Lake, Wisconsin	Hagerthey and Kerfoot^104^	–	Oligomesotrophic	0.001–0.065	0.0007–0.0455	SRP
Lake Hampen, Denmark	Ommen et al.^105^	–	Oligotrophic	0.004–0.05	0.024–0.3	SRP at 10–50 cm
Narrow Lake, Alberta	Shaw et al.^20^	2.6	Mesotrophic	0.175	0.063	TDP-Point estimate
Lake Mendota, Wisconsin	Brock et al.^106^	–	Eutrophic	0.27	0.486	Soluble PO_4_^–3^ at 4 cm
Lake Erie Western Basin, Ohio	Knights et al.^67^	–	Mesotrophic	0.12 (0.01–0.3)	0–12.94	TDP at 25 cm
Pine Barrens Ponds, NY	Schneider^26^	11.05	Oligotrophic	0.01	0.06–0.17	SRP at 10&50 cm
Oneida Lake—CBFS	Schneider et al.^41^	1.72	–	–	–	–
Lower Sylvan Pond, NY	Sebestyen and Schneider^52^	0.05	–	–	–	–
Devils Lake, Wisconsin	Lillie and Barko^49^	3.4	–	–	–	–
Lake Hampen, Denmark	Kidmose et al.^51^	15	–	–	–	–
Long Lake,Minnesota	Menheer^50^	130	–	–	–	–
108 lakes	Rosenberry et al.^58^	7.4 (0.05–1140)	–	–	–	–
Oneida Lake, South Shore	This study (Intensive)	1.05 (-1.6–23)	Mesotrophic	0.17 (0.004–2.22)	0.12 (0–2.41)	SRP at 30–40 cm
Oneida Lake	This study (Extensive)	0.77 (-0.9–5.12)	Mesotrophic	0.09 (0.01–0.85)	0.06 (-0.002–0.91)	SRP at 30–40 cm

*Single values represent means, while values in parenthesis represent a range (min–max).

**Type of P analyzed and sample depth collection.

Our observations agree with other studies that observed high spatial variability in seepage fluxes among sampling stations^53,54^. The observed variability in flow rates may be due to precipitation patterns acting at several spatial scales. In our intensive study, flow rates were positively correlated with the amount of rain in the previous 24 h, while in the lake-wide study there was a delayed effect of precipitation as flow decreased significantly with time since last rain event but was not correlated with rain volume. The discrepancy could be related to the scale of the precipitation data, as the 2018 study monitored rain daily within 150 m of sampling sites, while for the lake-wide study, the NOAA data sites were located 8–15 km away. Evidence suggests that the scale of the precipitation events could affect the length of the flow pathways activated, which will directly influence the time scale of the observed response on seepage flow. At the largest scale, during summer 2020, sites located along the south shore exhibited average flow rates twice that of the north shore. This pattern is opposite to that documented 15 years earlier^41^ and is likely explained by a shift in average precipitation between the two extensive halves (~ 1700 km^2^) of Oneida Lake’s watershed. Normally, the northern sub-watershed receives higher total precipitation than the southern basin. However, in 2020, both mean and total precipitation in the southern sub-watershed was two times higher than the precipitation in the northern sub-watershed^55^. Hydrometeorological conditions, both immediate and past, are known for having a big influence on subsurface flow magnitude and timing^56,57^. Rosenberry et al.^58^ reported a variety of time scales, from minutes to days, in the timing and magnitude of groundwater seepage flow rate responses to rain events measured at different scales, in eight lakes located across the United States. It is likely that local precipitation has a rapid effect on shallow local pathways measured in the 2018 study, while regional precipitation has a delayed effect, as in the lake-wide study, on shoreline flow rates as the pathways activated are longer and deeper^59,60^.

Adjacent land use was also a strong predictor of flow rates along Oneida Lake’s shoreline. During the intensive study, the Field site exhibited significantly higher rates, while the Wetland site exhibited the lowest and more constant flow. Our results suggest that the Wetland area is retaining or transpiring water during the summer at Oneida Lake, a process that has been reported for other natural and constructed wetlands^61,62^. During the lake-wide study, sampling sites located next to Forested areas exhibited significantly higher seepage rates compared to sites located adjacent to Residential areas. Residential areas are known to decrease the infiltration rate and increase the overland flow rates^63,64^. Studies that have evaluated the impacts of land use change on infiltration rate and groundwater recharge are consistent with our results^65,66^.

Most significant were groundwater seepage P concentrations and the resulting loads. In the lake-wide study, SRP concentrations ranged from 0.07 to 0.85 mg/L. Similar SRP concentrations were reported by Knights, et al.^67^ along Lake Erie, by Shaw, et al.^20^ for Lake Alberta in Canada, and in other U.S. lakes^52,68^. Much lower SRP concentrations were detected in pore waters of the oligotrophic ponds of Pine Barrens in Long Island, New York^26^ (Table 4). In 2018, the highest SRP concentrations were associated with Residential sites with active septic systems, contrasting with the lake-wide study where Residences were connected to the sewer system, and had relatively lower concentrations (max = 0.9 mg/L). Regardless, LLM results for the lake-wide study indicated significantly higher SRP concentrations in the sites located next to Residential areas compared to other sites; suggesting that even when sewer systems are installed, there might be a lag time in nutrient loading response after septic decommission^69^. Lakeshore residential development may also alter the flow and nutrient dynamics dramatically due to increased impervious area or lawn fertilization which can be significant where subsurface fluxes move rapidly into the lake^64,70^. These results suggest that the sites adjacent to areas with active septic systems could be acting as a hot spot^71^ or as a permanent control point^72^ for P export through groundwater seepage.

Combining flow rates and concentrations, SRP loads into the entire lakeshore were relatively low and constant, ranging from − 0.03 to 0.91 mg/m^2^-day, which is comparable to the values reported for the intensive south shore, and within the range for SRP loads reported for other lakes where similar methodology was used to estimate groundwater P fluxes (Table 4). In contrast, TP concentrations and loads were significantly higher and widely variable across time and space. TP concentrations during 2020 ranged from 0.17 to a maximum of 102 mg/L (x = 25.2 mg/L, SD = 29). As aquatic research typically focuses on analyzing dissolved P fractions, especially phosphate **r**eports of TP concentrations in groundwater seepage flowing directly into lake shorelines are rare. In this study SRP concentrations averaged less than 10% of TP (Table 3), indicating that organic dissolved P is likely the predominant fraction entering the lake via groundwater seepage. The sponge filter used in the porewater samplers should prevent coarse and fine size sediments grains**,** that may contain bound P**,** from entering the water (See methods section: water sampling and analysis). This unexpected finding has huge implications for P and primary productivity dynamics in lake shorelines, especially in the context of nearshore eutrophication and Harmful Algal Blooms. The organic fraction of TP has rarely been considered in traditional approaches dealing with excess P loading into lacustrine environments, despite evidence showing it is accessible by phytoplankton and bacteria. Prestigiacomo, et al.^73^ showed that the bioavailable fraction of dissolved phosphorus compounds ranged between 62 and 84% in a temperate northeastern U.S. lake. Furthermore, studies on cyanobacteria physiology suggest the existence of various physiological strategies to use dissolved organic phosphorus. These strategies include the utilization of extracellular enzymes such as phosphatase^47,74^ which are correlated with low concentrations of dissolved inorganic P^75,76^, as occurs during cyanobacterial blooms^77^. Moreover, laboratory experiments showed that some cyanobacteria species were able to sustain growth rates solely on organic P sources^78,79^. Xie et al.^74^ explored the physiological behavior of *Microcystis aeruginosa*, one of the leading cyanobacteria species in toxic algal blooms and demonstrated its ability to utilize organophosphates. Cumulatively, these results suggest that the structure and functions of phytoplankton community may rely not only on P concentrations but also on its composition, highlighting the potential importance of groundwater P sources to lakes.

Evaluation of the relationships between P patterns, land use, and precipitation provides some additional insights into the potential biogeochemical processes of TP in lakes. During summer 2020, precipitation volume in the previous 72 h was correlated with increasing TP concentrations, suggesting that rain events are leaching the TP out of the adjacent watershed into the lake (Table S2, Fig. S1). Moreover, adjacent shore land use did not show an effect on TP concentrations, which raises the question of the source of this phosphorus. One possible explanation underlying the high concentrations of organic P, could be related to the reported increases of dissolved organic matter (DOM) loading that many lakes in North America and Europe have experienced in recent years^80,81^. This phenomenon, labeled as “browning,” has a significant impact on carbon (C) cycling and affects a range of physical and ecological attributes and processes within lakes. However, studies suggest that it may influence the loading of other nutrients to lakes^82,83^. Studies of leachates from different terrestrial materials showed that DOM contained different amounts of N and P, and in some cases, it can greatly impact N and P fluxes to lakes^48,84^. Corman et al.^85^ documented significant relationships among DOC, P and Nitrogen (N) loadings into seepage lakes in Wisconsin, concluding that nutrients associated with DOM may substantially influence N and P concentrations in lakes. Increasing DOC loading has been documented in lakes in the northeastern US even where it is not always associated with a change in color^86^, especially in systems with short residence times, like Oneida Lake^87^. More research is urgently needed to understand the links between C and P cycles, and the potential role of groundwater seepage connecting terrestrial with aquatic biogeochemical processes.

There was clear evidence of nearshore P enrichment throughout the entire shoreline of Oneida Lake. Porewater P concentrations were significantly higher than those of the associated surface water samples, and concentrations of P in shoreline surface waters were significantly higher than offshore open lake concentrations. Groundwater-surface water gradients in P concentrations have been reported in many temperate lakes and the underlying mechanisms are still under investigation and discussion. Extensive research was conducted in Lake Ontario^36^, and other Great Lakes^38,88^, with intriguing results and postulations about the various roles of different factors such as stream discharge, hydrodynamics, and temperature entrapment. Our findings indicate that groundwater seepage is a significant contributor of P into lakeshores and this source of P is widely overlooked. It identifies special importance for the littoral environment as groundwater seepage interacts in the nearshore where shallow pathways directly discharge into lakes. Moreover, its relevance increases in the context of shore benthification processes that have been documented in many temperate lakes after dreissenid mussels’ invasions^89–91^.

Finally, groundwater TP loads around the entire Oneida Lake shoreline exhibited high and widely ranging values but comparable to those reported for internal loading in other temperate lakes^45,46^, but this loading is occurring at the sediment–water interface throughout the entire lake shoreline. Indeed, it is possible that a portion of groundwater loading may have been included in internal loading estimations for P budgets, as its differentiation is empirically very challenging, and studies keep excluding one from the other, albeit acknowledging the potential relevance of both^6,46^. From a management perspective it is crucial to differentiate between the two, as internal loading is related to legacy P, while groundwater seepage is a new, external P source. The groundwater loads were also comparable to those from Oneida’s tributaries^44^. Lisboa^44^ conducted an extensive analysis comparing P loading into Oneida Lake from tributaries vs. groundwater during summer 2020. Cumulative loading estimations from the five main tributaries into the lake added up to 4744 kg of TP, while groundwater loading throughout a 20 m wide belt around the entire lakeshore was estimated at 3335 kg of TP during the same six summer weeks. Moreover, if only baseflow conditions (excluding storm flow events, when the majority of sediment bound TP runoff occurs) are considered, cumulative TP loading from streams decreases to 2219 kg (Fig. S2). Other estimates of P loading from tributaries fall within a similar range, such as Makarewicz and Lewis^92^, whose estimates of TP loading from the same 5 tributaries over the growing season 2002 (March to September) added up to 6216 kg of TP. These figures are driven by the high P content of the inflowing groundwater, as groundwater flow inputs have been estimated to represent around 3% of the annual water inflow into Oneida lake^87^.

Spatial heterogeneity has been previously recognized as one of the major challenges related to the study of chemicals transported by groundwater^6,93^. In addition, these results highlight the need for further investigation as groundwater seepage loadings may have significant implications for lake nutrient dynamics and trophic conditions. In our study, TP is dominated by the soluble fraction, with likely little to no particulate fraction, which means that most of the P entering the lake via groundwater seepage may be available for biological consumption. As a result, even if groundwater seepage loading represents a small fraction of the total P loading to the lake, it may be highly significant for the trophic states of the lake shore, considering its high relative bioavailability. We argue that groundwater P transport may be a driving factor in many lakes globally and needs to be investigated and addressed to predict and understand the response of lake ecosystems to nutrient management.

## Methods

### Study area

**Figure 4:**
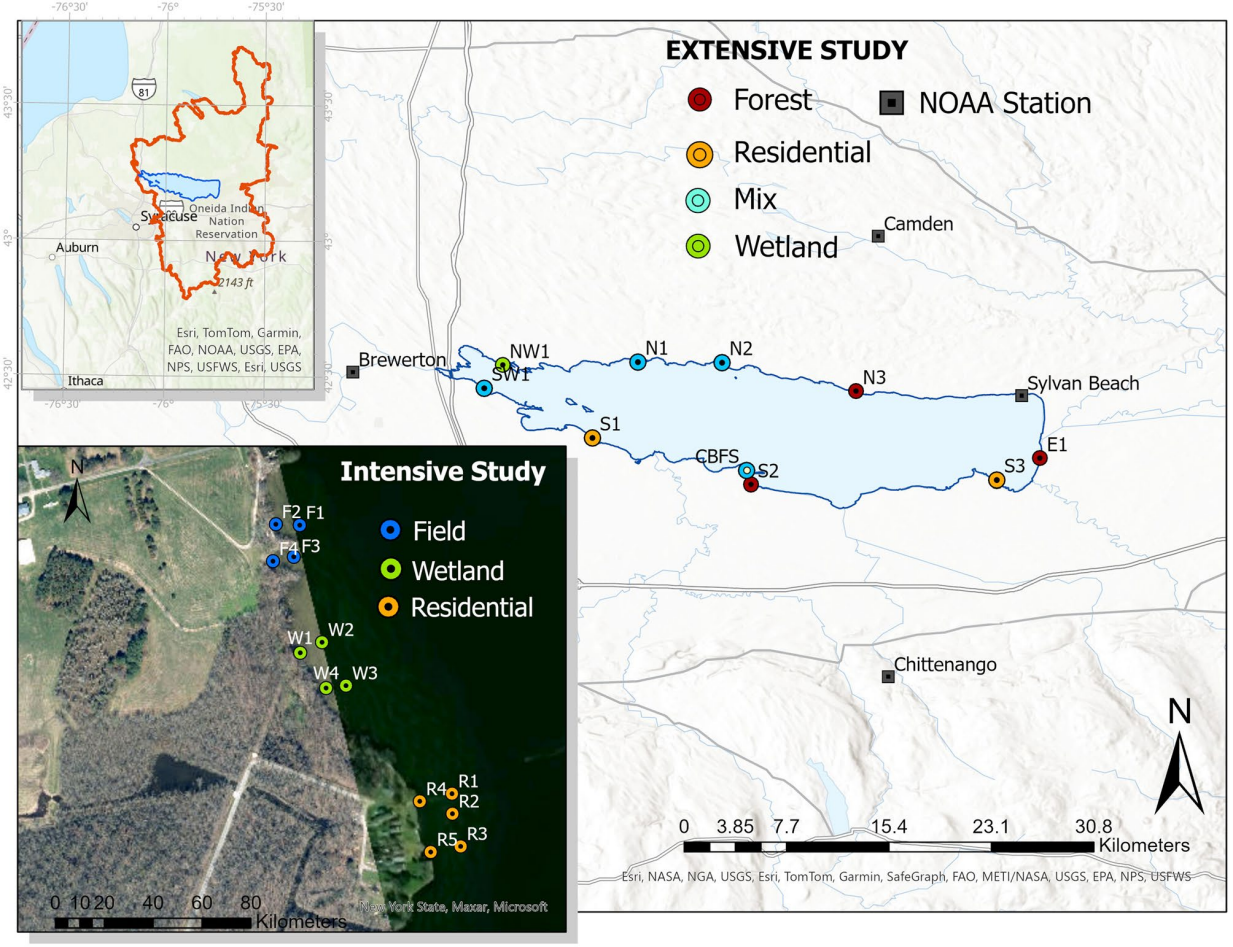
Oneida Lake location within NY State, and its drainage basin with the watershed divide indicated with a red line in the upper left map. Sampling stations (points) for the intensive and extensive study are shown in the lower left and the main map, respectively. Colors represent shore adjacent land use. NOAA stations refer to weather stations used for precipitation analysis. This figure was created using stellite images in ArcGIS Pro 3.0.0. (ESRI (2022) ArcGIS Pro: release 3.0.0. Environmental Systems Research Institute., Redlands, CA. https://www.esri.com/en-us/arcgis/products/arcgis-pro/overview).

Our study was conducted at Oneida Lake (43° 10′ N, 75° 52′ W), located in central New York, 18 km east of the city of Syracuse. The lake is part of the Oswego River Basin which is part of the greater Lake Ontario basin. The lake is of glacial origin, with a consolidated sedimentary bedrock underlying the whole basin^91^. The watershed is characterized by a continental climate with warm, dry summers and cold, snowy winters. The lake runs from east to west dividing its 3579 km^2^ watershed into two similar sized halves, with long North and South shores associated with the two halves (Fig. 4). The northern and southern sub-watersheds are of similar geological origin, both presenting geological layers with high hydraulic conductivity, but prominent differences in its hydrometeorological conditions, which have significantly influenced its land use and land cover. The southern half underlying geology consists of successive bedrock layers of limestone, shale, dolomite, and sandstone overlain by unconsolidated glacial till. The northern half is dominated by a 76 m thick base layer of sandstone shale, which intercepts and underlies a portion of Oneida Lake, and is overlain successively by layers of dolomite, middle shale, limestone and upper shale^94^. The mean annual precipitation is 965 mm in the southern basin and 1300–1500 mm in the northern basin^95^. Much of the precipitation originates as snowfall in the winter and is stored as snowpack. The northern sub-basin received 3–5 m of snowfall annually, which is the highest reported snowfall east of the Rocky Mountains, due to the basin position east and down-wind of Lake Ontario. As a result of the long snowy winters and acidic soils, the northern half is dominated by forest with numerous wetlands, and minimal agriculture; while the southern half is dominated by agriculture, suburban areas around the city of Syracuse, woodlands, and a 2100 ha swamp (Cicero Swamp)^87^. Approximately 56 percent of the precipitation that falls in the watershed reaches the lake through surface inflow^96^. The rest is redistributed through evaporation, transpiration by trees and plants, and groundwater recharge. Seven tributaries, three in the south, four in the north, contribute to the overland surface flow to Oneida Lake. Although surface inflow from the northern watershed represents most of the total water volume entering the lake, the majority of the nutrient inputs are coming from tributaries that drain the farmlands of the southern watershed. Water flows out of Oneida Lake into Oneida River which is located at the western edge of the lake. The combined sedimentary bedrock and glacial deposits result in a high ability to store and transmit water creating an extensive system of aquifers that underly large portions of the watershed. This setting, combined with a steep topographic relief over hundred meters of elevation towards Oneida Lake, facilitates the superficial and groundwater flow from both sub-basins into Oneida Lake^85^.

The watershed includes portions of six counties and 69 municipalities, with a human population of 262,164 predominately concentrated in Onondaga County according to the 2000 US Census. The 2020 US Census data did not reveal a significant change in population within the counties compromising the watershed area. The lake is extensively used for recreation, fishing, and general tourism, playing a large role in the local economy. The lake’s shoreline extends for 88 km, is mainly used for recreational activities, and is characterized by both permanent and seasonal housing. Approximately 72% of the housing units in the Oneida Lake watershed are serviced by a public sewer system, with the remaining housing relying on septic systems for wastewater disposal. Most of the houses on the lake shoreline have been connected to a sewer system with a few houses still using septic systems. Changes around Oneida lake are being made annually, but at the time of this research, portions of the study shoreline were still using septic systems.

### Sampling sites and design

Over the course of three summers, we conducted two studies focusing on different scales along Oneida Lake. First, during summer 2017 and 2018, we focus our research on a selected portion of shoreline located in the southern basin, which we refer to as the intensive study. In a second phase, which took place during summer 2020, we monitor and sampled 10 representative sites located around the entire shoreline of Oneida Lake which we refer to as the extensive study. For the intensive study during the summers of 2017 and 2018, 11 & 13 sampling stations, respectively, were installed along 800 m southern shoreline of Oneida Lake, proximal to Cornell Biological Station (CBFS). and coincident with reference sites used in previous studies^41^. The area allowed us to sample from the three dominant shore land uses in the watershed, with easy and convenient access to CBFS. The shoreline adjacent land use to each sampling site was identified using Google EarthPro by categorizing the main land cover in a rectangle of 200 m parallel to the shore by 500 m wide in the shore area adjacent to the sampling site. If more than 5 houses were located on the shoreline, the site was characterized as residential despite other land covers. The first section has ~ 260 m of shoreline adjacent to an abandoned agricultural field, followed by ~ 300 m of shoreline associated with a red maple swamp forest, which in turn was followed by 140 m of shore associated with nine houses (eight seasonal, one permanent) located within 50 m of the lakeshore edge (Fig. 4). At the time of this study, none of these houses were connected to a sewer line, but instead relied on septic systems.

For the Lake-wide study, we selected 10 sites distributed along the entire shoreline of Oneida Lake to install sampling stations for groundwater seepage flow and its P concentrations. Sites were selected for its accessibility from land or by boat and were chosen to get representation of the whole lake shoreline, which result in five sites located along the southern shore including the reference site from the local and previous studies, four sites along the northern shore, and one site on the eastern shore in between the two main tributaries inlets (Fig. 4). Shoreline adjacent land uses analysis resulted in four sites located next to forested areas, two in residential areas, four that were a mix of residential, forest and abandoned field, and one site next to a wetland area.

Sampling stations were identical for both studies and consisted of a seepage meter modified from the design of Lee^97^, with the use of a plastic cover to reduce wave impact^41^; and a porewater sampler^26^ installed to a depth of ~ 40 cm into the lake sediments. The pore water samplers used in this study were designed by Dr. Schneider at Dr. Howarth’s Lab at Cornell University in the early 90’ and have been used to understand groundwater seepage solute concentrations in small freshwater lakes^26,52,98^.

For the intensive study sampling stations were equally distributed among the three land uses at two different distances from shore (10 and 38 m). This design was chosen to be consistent with previous studies from the early 2000s^41^ and thus facilitating comparisons. Seepage meters were monitored three to four times a week during the 2017 and 2018 seasons, and pore and lake water samples were collected once to twice a week during 2018. Lake samples were taken in the immediate vicinity of pore samplers with the purpose of comparing pore and lake water chemistry with a focus on P concentrations.

For the extensive study sampling stations were located 10 m from the shore only. Lake samples were also taken in the immediate vicinity of pore samplers with the purpose of comparing pore and lake water chemistry with a focus on P concentrations. Samples were taken once a week for nine weeks during summer 2020 (June to August). Groundwater seepage meters were activated 24–72 h. before sample collection and reading. Due to accessibility issues and personnel availability restrictions associated with the COVID-19 pandemic, not every site was sampled every week, which resulted in a heterogeneous number of samples at each site (Table 3). Sites located on the southern and eastern shore were more accessible due to the availability of land access. Consequently, samples from these sites were collected more or less on a weekly basis (n = 9–14), except for site S3, which was added later in the season. On the other hand, sites located on the northern shore were more challenging to access, resulting in fewer samples from each site, except for site N2, where samples were collected weekly (n = 10). However, for the remaining northern sites (n = 5–6), we ensured sample collection throughout the entire season, with a minimum of one sample per month at each site. At Site NW1 located next to a big wetland, seepage meters were consistently missing the day after installation for unknown reasons (removed by the public, high water flow, loose soil). As a consequence, there is no seepage reading for this site, and in order to estimate P loadings using P concentrations sampled for that site, we used the weekly average flow rates for sites located on the north shore.

### Water sampling and analysis

Groundwater seepage was estimated at each site using seepage meters following the method established by Lee^97^. Each bag was initially filled with 500 ml of water to avoid artificial bag inflation problems, and to allow for monitoring of recharging flow^41^. The bags remained attached to the meters for at least 24 h and up to 72 h. After that time, the bags were removed, and volume change was determined using a graduated cylinder. Increases in volume indicated groundwater discharge into the lake, while volume loss indicated lake water recharging into groundwater. The seepage bags were replaced on a weekly basis to avoid over-expansion or leakage. Data was not incorporated in the analysis if either bags or meters covers showed signs of damage. Groundwater flow was calculated using the following equation:

1 $$Q=\frac{\Delta V}{Axt}$$

where, Q: Seepage flow rate, $\Delta$V: seepage bag volume change, A: Surface area covered by the seepage meter, t: Time.

During 2018 and 2020, pore and lake water samples were collected for Total Phosphorus (TP) and Soluble Reactive Phosphorus (SRP) analyses. Before sample collection, pore samplers were first flushed two times when enough flow was available (~ 50 ml); however, toward the drier period of the summer only the first 20 ml of sample were discarded to ensure new water flowing into the device was collected. Water samples were collected in acid washed 60 ml poly-propylene bottles, stored at 4 °C in a cooler, and transported to the nearby Cornell Biological Field Station laboratory. Once in the lab, each sample was divided into two portions for the various analyses. Approximately 15 ml were immediately filtered through a 0.45 μm filter (Supor Membrane Disc Filter, 25-mm diameter) and stored for SRP analysis. Additional unfiltered 25 ml subsamples were stored separately for TP analysis. All the samples were stored at 4 °C. To halt potential microbial cycling of nutrients, we decreased the pH to 2 by adding 30% H_2_SO_4_ before storage^99^. After collection and preservation, nutrient analyses were run within one year at the Soil and Water Laboratory at Cornell University located in Ithaca, New York. Sample pH was checked monthly to ensure sample preservation. Phosphorus analysis was done on an automated wet chemistry analyzer (FS3000; Xylem Analytics O.I. Analytical, Beverly, Massachusetts) screening for phosphate anions (PO_4_^3−^) in SRP and TP samples. TP samples were first digested with persulfate and sulfuric acid^96^ and then filtered through a 0.45 μm filter (Supor Membrane Disc Filter, 25-mm diameter). Reagents for analysis were ammonium molybdate, ascorbic acid, sulfuric acid, and potassium antimonyl tartrate^99^. Each run was calibrated using 0.05, 0.1, 0.5, 1 and 5 ppm potassium phosphate standards, with all standard curve R^2^ values between 0.998 and 0.999. If a given sample’s P concentration was above the calibration range, the sample was diluted to ensure the quality of the data. It is important to note that even though we analyzed the samples for TP and not for Total Dissolved P (TDP), meaning we did not filter the samples in the laboratory before digestion, most of our samples were clear in color. The permeable tips of the pore water samplers (as detailed in the previous section) was perforated to ~ 1 mm in diameter, and the flexible plastic tube inside was wrapped in a polyethylene sponge filter, with the purpose of preventing small clay sized sediments from interfering with sampling. In addition, for the local study, during 2018, we measured dissolved oxygen concentrations (MW600; Milwaukee Instruments) in the lake and the pore water samples, directly in the field at the time of collection. Lake DO concentration was measured by inserting the probe in the top 10 cm of the lake water; for pore samples the DO probe was inserted in a special port created in the syringe used to collect the sample as soon as possible after suction to minimize sample oxygenation.

### Data and statistical analysis

For the data analysis we first use a descriptive summary analysis to characterize and estimate groundwater flows, P concentrations, and P loads pattern into Oneida Lake shoreline. P loads were calculated by multiplying P concentrations by average hourly groundwater seepage flow in the period between sample collection. For flow analysis we used the data collected during 2017, 2018 and 2020, while for concentrations and loads we used the data collected during 2018 and 2020, for the intensive and extensive study, respectively.

In addition, we used linear regression modelling to evaluate relationships between potential controlling factors, including precipitation and shore adjacent land use and observed groundwater seepage flow, P concentration, and P loads. In addition, for the extensive study models were also used to compare flows, concentrations, and loadings between the north and the south shore. Given the studies differing scales precipitation data used for each analysis also differ in its spatial and temporal scale. For the intensive study we used the amount of local precipitation in the previous 24 h, which was monitored daily on site using a manual rain gauge located 150 m from the south shoreline in an open field. For the extensive study, precipitation data was obtained from the closest NOAA weather station to each sampling site (8–15 km away), which resulted in more than one precipitation data set involved in the analysis, and in most cases one station was used for more than one site (Supplementary Table S3). Thus, for precipitation analyses a random effect for weather station was also included in the models. Seven aggregations of the precipitation data were used for this analysis (Precipitation in the previous 24 h, 48 h, 72 h, cumulative precipitation in the previous 48 h and 72 h, and number of days since the last rain event), in order to understand how regional rainfall affects groundwater seepage flows and its P loading at the lake shoreline. Each precipitation aggregation was used separately, resulting in seven different models for each response variable, with the final model selection based on Akaike's information criterion (AIC)^100^. Also, for the lake wide study analysis, the site located on the east shore (E1) was excluded from the shore analysis; and the site located next to the wetland area (NW1) was excluded from the land use analysis due to lack of replication.

All response variables were log_e_ transformed to meet normality assumptions, and predictor variables with large absolute values were scaled in order to avoid inflated coefficients. In order to account for repetitive measurements over time at the same site we used linear mixed effect models (LMM) fitted by maximum likelihood using site as a random effect for the intercept. In addition, due to the longitudinal structure of the data set, we included time as a fixed effect^101^. As a result, we used the following null model for all the response variables:

$$ {\text{Response variable}}\sim {\text{Time}} + \left( {{1}|{\text{Site}}} \right). $$

For each response variable, several LMM were developed using a forward selection approach. The final model selection was based on Akaike's information criterion (AIC)^100^, and sequential F ratio testing^102^. Diagnostic plots of best-fit model residuals did not show major deviations from the assumptions of normality and constant variance. In addition, to understand the differences within categorical variables, we used a Type III Analysis of variance (ANOVA) using the Satterthwaite approximations to degrees of freedom.

Results were considered significant when p-values < 0.05. All statistical analyses were run in R software (R Core Team 2021). Mixed models were run using the lmerTest package^103^.

### Supplementary Information

Supplementary Information.

## Data Availability

The datasets generated during and analyzed during the current study are available from the corresponding author on reasonable request.
