# Supplementary material for: Groundwater inputs could be a significant but often overlooked source of phosphorus in lake ecosystems
Source: Sci Rep. 2024 Jul 15;14:16269. doi: 10.1038/s41598-024-66985-z (PMC11251285; doi:10.1038/s41598-024-66985-z)
Supplement: Supplementary file 1 — Supplementary Information. [file 41598_2024_66985_MOESM1_ESM.docx]

Table S1. Fixed effects models estimates for precipitation and land use analysis for intensive study. P-values estimated via t-test using Satterthwaite approximation to degrees of freedom. Estimates are in natural logarithmic scale. Significant p-values are bolded.

| Response Variable | **Groundwater Flow (ln Flow (L/m^2^ h)** | | | |
| --- | --- | --- | --- | --- |
| Model | Flow ~ Precipitation + Land use + Time + (1\|Site) | | | |
| *Predictors* | *Estimates* | *SE* | *t* | *p* |
| (Intercept) | 3.17 | 5.65 | 0.56 | 0.575 |
| Time | -0.12 | 0.13 | -0.89 | 0.374 |
| Precipitation (mm) | 0 | 0.001 | 1.83 | 0.068 |
| Wetland Land use | -0.26 | 0.06 | -4.46 | **<0.001** |
| Residential Land use | -0.14 | 0.06 | -2.42 | **0.016** |
| Response Variable | **SRP concentration (ln SRP (mg/L))** | | | |
| Model | SRP ~ Time + (1\|Site) | | | |
| *Predictors* | *Estimates* | *SE* | *t* | *p* |
| (Intercept) | -216.07 | 122.97 | -1.76 | 0.081 |
| Time | 49.37 | 28.4 | 1.74 | 0.084 |
| Response Variable | **SRP Load (ln SRP (mg/m2 h))** | | | |
| Model | SRP Load ~ Precipitation + Land use + Time + (1\|Site) | | | |
| *Predictors* | *Estimates* | *SE* | *t* | *p* |
| (Intercept) | -57.77 | 114.32 | -0.5 | 0.614 |
| Time | 1.23 | 2.64 | 0.47 | 0.641 |
| Precipitation mm | 0.01 | 0.003 | 2.18 | **0.031** |
| Wetland Land use | -0.32 | 0.13 | -2.47 | **0.015** |
| Residential Land use | -0.1 | 0.13 | -0.82 | 0.412 |
| Response Variable | **TP concentration (ln TP (mg/L))** | | | |
| Model | TP~ Time + (1\|Site) | | | |
| *Predictors* | *Estimates* | *SE* | *t* | *p* |
| (Intercept) | -1019.91 | 208.22 | -4.9 | **<0.001** |
| Time | 235.56 | 48.1 | 4.9 | **<0.001** |
| Response Variable | **TPLoad (ln TP (mg/m2 h))** | | | |
| Model | TP Load ~ Land use + Time + (1\|Site) | | | |
| *Predictors* | *Estimates* | *SE* | *t* | *p* |
| (Intercept) | -1055.26 | 461 | -2.28 | **0.024** |
| Time | 242.95 | 106.6 | 2.28 | **0.025** |
| Wetland Land use | -1.48 | 0.47 | -3.15 | **0.002** |
| Residential Land use | 0.07 | 0.47 | 0.15 | 0.882 |

Table S2. Fixed effects models estimates for precipitation and land use analysis for extensive study. P-values estimated via t-test using Satterthwaite approximation to degrees of freedom. Estimates are in natural logarithmic scale. Significant p-values are bolded.

| **Precipitation Analysis** | | | | |
| --- | --- | --- | --- | --- |
| Response Variable | **Groundwater Flow (ln Flow (L/m^2^ h)** | | | |
| Model | Flow ~ #DryDays + Date + (1\|Site) + (1\|NOAAStation) | | | |
| *Predictors* | *Estimates* | *SE* | *t* | *p* |
| (Intercept) | 40.83 | 119 | 0.34 | 0.734 |
| Date | -0.001 | 0.002 | -0.36 | 0.718 |
| Dry Days (#) | -0.07 | 0.03 | -2.62 | **0.01** |
| Response Variable | **TP concentration (ln TP (mg/L))** | | | |
| Model | TP ~ Precipitation + Date + (1\|Site) + (1\|NOAAStation) | | | |
| *Predictors* | *Estimates* | *SE* | *t* | *p* |
| (Intercept) | 1819 | 229.7 | 7.91 | **<0.0001** |
| Date | -0.041 | 0.005 | -7.91 | **<0.001** |
| Precipitation 72hs (mm) | 0.67 | 0.26 | 2.51 | **0.01** |
| Response Variable | **TP Load (ln TP (mg/m2 h))** | | | |
| Model | TP Load ~ Precipitation + Date + (1\|Site) + (1\|NOAAStation) | | | |
| *Predictors* | *Estimates* | *SE* | *t* | *p* |
| (Intercept) | 1462 | 592 | 2.47 | **0.02** |
| Date | -0.03 | 0.01 | -2.47 | **0.02** |
| Precipitation mm | 1.19 | 0.69 | 1.74 | *0.09* |
| Response Variable | **SRP Load (ln TP (mg/m2 h))** | | | |
| Model | SRP Load ~ Precipitation + Date + (1\|Site) + (1\|NOAAStation) | | | |
| *Predictors* | *Estimates* | *SE* | *t* | *p* |
| (Intercept) | 74.35 | 139 | 0.53 | 0.59 |
| Date | -0.001 | 0.003 | -0.57 | 0.56 |
| Dry Days (#) | -0.063 | 0.029 | -2.13 | **0.036** |
| **Land use analysis** | | | | |
| Response Variable | **Groundwater Flow (ln Flow (L/m^2^ h)** | | | |
| Model | Flow ~ LandUse + Date + (1\|Site) | | | |
| *Predictors* | *Estimates* | *SE* | *t* | *p* |
| (Intercept) | 44.67 | 123 | 0.36 | 0.71 |
| Date | -0.001 | 0.002 | -0.38 | 0.70 |
| Residential | -0.75 | 0.26 | -2.84 | **0.01** |
| Mix | -0.42 | 0.21 | -1.97 | *0.08* |
| Response Variable | **SRP concentration (ln SRP (mg/L))** | | | |
| Model | SRP ~ LandUse + Date + (1\|Site) | | | |
| *Predictors* | *Estimates* | *SE* | *t* | *p* |
| (Intercept) | 317 | 135 | 2.33 | **0.02** |
| Date | -0.007 | 0.003 | -2.35 | **0.02** |
| Residential | 0.42 | 0.18 | 2.28 | **0.02** |
| Mix | 0.107 | 0.14 | 0.74 | 0.46 |
| Response Variable | **TP Load (ln TP (mg/m2 h))** | | | |
| Model | TP Load ~ LandUse + Date + (1\|Site) | | | |
| *Predictors* | *Estimates* | *SE* | *t* | *p* |
| (Intercept) | 1775 | 542 | 3.27 | **0.001** |
| Date | 0.04 | 0.012 | -3.27 | **0.001** |
| Residential | -2.59 | 1.08 | -2.4 | **0.03** |
| Mix | -1.12 | 0.88 | -1.28 | 0.23 |
| Response Variable | **SRP Load (ln SRP (mg/m2 h))** | | | |
| Model | SRP Load ~ LandUse + Date + (1\|Site) | | | |
| *Predictors* | *Estimates* | *SE* | *t* | *p* |
| (Intercept) | 110 | 141 | 0.78 | 0.44 |
| Date | -0.002 | 0.003 | -0.82 | 0.42 |
| Residential | -0.52 | 0.29 | -1.77 | 0.107 |
| Mix | -0.44 | 0.24 | -1.84 | *0.09* |

Table S3. Meteorological data used for precipitations analysis in the extensive study. NOAA Stations Name and ID and its association with groundwater sampling sites.

| **NOAA Station ID** | **NOAA Station Name** | **Sampling Sites** |
| --- | --- | --- |
| US1NYMD0016 | CHITTENANGO 2.1 ESE, NY US | S1, S2, S3, CBFS |
| USC00301110 | CAMDEN, NY US | N1, N2, N3 |
| US1NYOD0061 | SYLVAN BEACH 1.6 NW, NY US | E1 |
| USC00300870 | BREWERTON LOCK 23, NY US | NW1, SW1 |


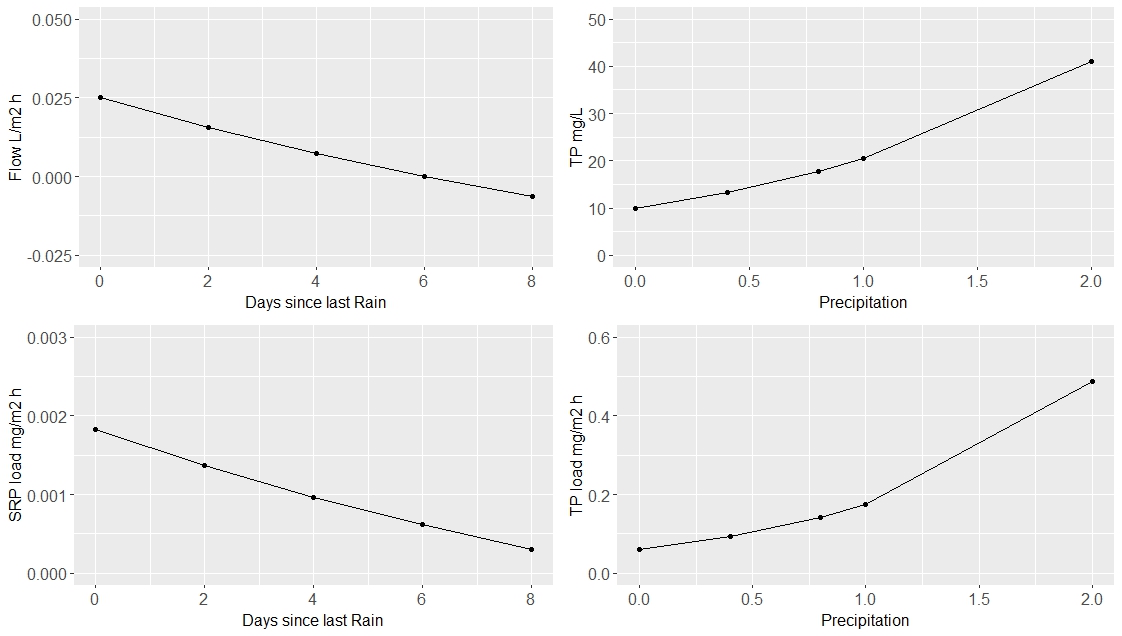


**D.**

**C.**

**B.**

**A.**

Figure S1. Flow, concentrations, and loads estimates in response to precipitation. A. Groundwater flow rate response to number of days since last rain event. B. TP concentrations response to precipitation (mm) in the previous 72 hrs. C. SRP load response to number of days since the last rain event. D. TP load response precipitation (mm) in the previous 72 hrs.


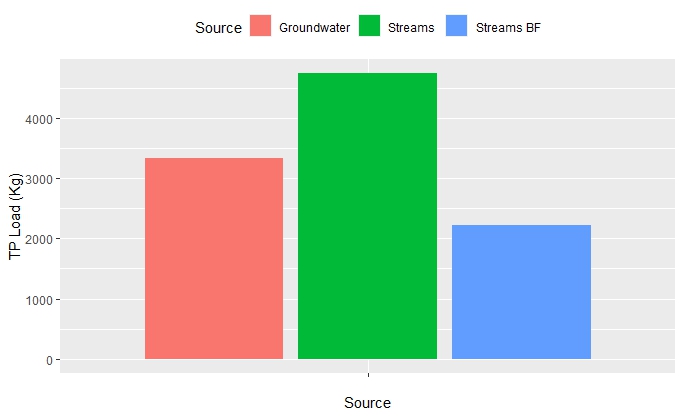


Figure S2. Estimated cumulative TP load (kg) from streams (green bar) vs. groundwater (red bar) into Oneida lake over six weeks during summer 2020. Streams loads were also calculated during baseflow conditions only excluding high flow events from the analysis (blue bar).
